# The Baltimore Medical College

**Published:** 1881-10

**Authors:** 


THE BALTIMORE MEDICAL COLLEGE.—From the many queries about our personal relation and that of the INDEPENDENT PRACTITIONER to this *new* institution, we deem it necessary, in justice to ourself, to state publicly that we have been *urged* from its conception to be a part of this new organization, and have constantly declined to be associated with its faculty. Neither the *journal* nor our influence will be used for this or any other individual college.

